# Role of Microglia in CNS Autoimmunity

**DOI:** 10.1155/2013/208093

**Published:** 2013-06-12

**Authors:** Tobias Goldmann, Marco Prinz

**Affiliations:** ^1^Institute of Neuropathology, University of Freiburg, Breisacher Straße 64, 79106 Freiburg, Germany; ^2^BIOSS Centre for Biological Signalling Studies, University of Freiburg, Breisacher Straße 64, 79106 Freiburg, Germany

## Abstract

Multiple sclerosis (MS) is the most common autoimmune disease of the central nervous system (CNS) in the Western world. The disease is characterized histologically by the infiltration of encephalitogenic T_H_1/T_H_17-polarized CD4^+^ T cells, B cells, and a plethora of myeloid cells, resulting in severe demyelination ultimately leading to a degeneration of neuronal structures. These pathological processes are substantially modulated by microglia, the resident immune competent cells of the CNS. In this overview, we summarize the current knowledge regarding the highly diverse and complex function of microglia during CNS autoimmunity in either promoting tissue injury or tissue repair. Hence, understanding microglia involvement in MS offers new exciting paths for therapeutic intervention.

## 1. Multiple Sclerosis: The Most Frequent Autoimmune Disease of the CNS

An autoimmune disease is characterized by the loss of self-tolerance of the immune system, which can be caused by either genetic or environmental factors or a combination of both [1]. As a consequence of this malfunction, an immune response is initiated against certain cell types or even entire organs of the body. For the central nervous system (CNS) several autoimmune diseases are described of which multiple sclerosis (MS) is the most common form, affecting approximately 2.5 million people worldwide, mainly in the third and fourth decades of live. While the exact etiology of MS is still unknown, much progress has been made in understanding its pathology. MS comprises a blood-brain-barrier (BBB) disruption accompanied by an activation of macrophages/microglia as well as T- and B-cell infiltration into the CNS, ultimately resulting in demyelination and degeneration of neuronal structures [2]. MS can be clinically divided into different forms. Most patients experience relapsing-remitting stage (RRMS) of the disease, which in many cases results in continuous disease progression called secondary progressive MS (SPMS). On the other hand, some patients suffer from primarily progressive MS (PPMS), characterized by a continuously progressing disease course [2]. To date no cure for any form of MS exists, but several treatment options which might reduce the symptoms are available [3]. One of these approaches compromises the application of IFN-*β*, which is thought to be anti-inflammatory in RRMS and thereby reduces the relapse rate [3–5]. Unfortunately, even though IFN-*β* is well tolerated by patients, approximately 50% of them respond to and benefit from the treatment [5]. The effects of IFN-*β* are complex and far from being fully understood. Profound insights into the pathogenic mechanisms involved in MS as well as possible therapeutic interventions were gained through the use of experimental autoimmune encephalitis (EAE), the most used animal model for CNS autoimmunity [6, 7]. Several key features of MS, such as paralysis, weight loss, demyelination, and inflammation, observed in human patients, are recapitulated during EAE in rodents [7]. Depending on the strain, EAE can be induced by active immunization with myelin derived proteins such as myelin oligodendrocyte glycoprotein (MOG), myelin basic protein (MBP), or proteolipid protein (PLP) in combination with an adjuvant, usually complete Freund's adjuvant (CFA) [7]. CFA contains inactivated mycobacteria and is thought to break peripheral tolerance, which results in the induction of CNS autoimmunity. CFA is recognized by pattern recognition receptors such as Toll-like receptors (TLRs). Especially, myeloid differentiation primary response gene (88) (MyD88), TLR7, and TLR9 have been found to be essential disease modifiers. In addition to these surface and endosomal receptors, newly discovered endosomal molecules such as retinoic acid inducible gene- (RIG-) I and melanoma differentiation-associated protein- (MDA-) 5 also have been shown to be crucial for EAE induction [8, 9]. Some of these disease modifying recognition receptors release type I interferons (IFNs) upon activation that in turn robustly change both the innate and adaptive arms of autoimmunity in mice [9–12]. However, for C57BL/6 mice the EAE model induced by MOG_35-55_ peptide is thought to be a monophasic chronically active disease without significant recovery and relapse phases, thereby only partially reflecting the clinical course found in MS patients [13]. Nevertheless, this EAE model in addition to the increasing availability of (cell type-)specific knock-out mice has greatly expanded our understanding of MS pathology and might open new avenues for specific treatment options in the future. 

## 2. Microglia-Resident Macrophages of the CNS

Microglia cells are resident tissue macrophages located in the CNS and are considered to be a patrolling immune competent cell type within the parenchyma [14–17]. Microglia make up around 10% of the cells in CNS and are evenly distributed in the parenchyma of a healthy brain [14]. In contrast to neurons and macroglia (oligodendrocytes and astrocytes), microglia originate from the primitive hematopoiesis within the yolk sac (YS) and migrate to the neuronal tube during embryogenesis [17, 18]. Recently, we could identify lin^−^-erythromyeloid precursors as the genuine microglia progenitors on a stem cell level [19]. These pioneer cells continue their physiological and morphological development on their way from the YS to the developing brain, where the mature cells finally reside and build the ultimate pool of microglia [17, 19, 20]. By using parabiotic mice, a contribution of bone marrow-derived phagocytes (BMDPs) to the pool of existing microglia throughout life could be ruled out [20–22]. In fact, BMDP engraftment from the circulation can only be achieved after significant “priming” of the host CNS, for example, by irradiation-induced changes of the BBB or alterations of the tissue micromilieu [19, 23–25]. 

**Figure 1:**
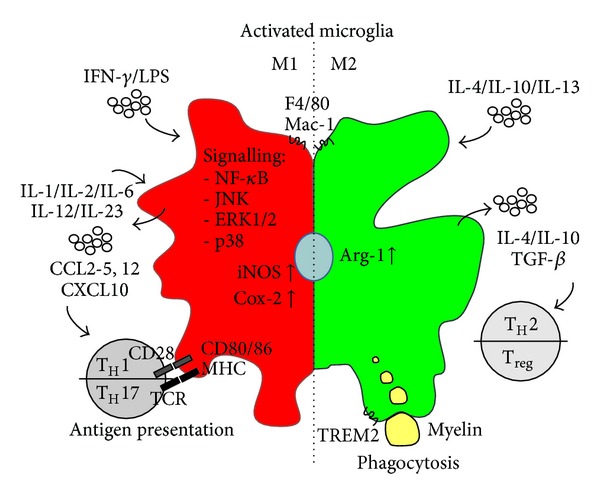
Polarization and function of activated microglia in CNS autoimmune inflammation. Microglia are activated by diverse stimuli, which define the polarization status of the cell. While IFN-*γ*/LPS promote the proinflammatory M1 status, IL-4/IL-10 or IL-13 induce the anti-inflammatory M2 status. M1 microglia take part in the attraction and differentiation of pathogenic T_H_1/T_H_17 T-cells, whereas M2 microglia promote phagocytosis of myelin debris, which is important for remyelination.

Due to their morphology, microglia cells are ascribed to be in a “resting” state under healthy conditions. This term is somewhat misleading, since *in vivo* imaging has revealed that microglia actively scavenge and monitor with their ramified branches the environment of the CNS for pathogens [26, 27]. Upon tissue damage, or inflammation, viral or bacterial insult, microglia change their morphology towards an amoeboid shape by retracting these branches. In addition to the morphological differentiation, several surface markers, such as F4/80 or Mac-1, which is typical for macrophages, are upregulated [28, 29]. The status of activation can be further subdivided into “classically” (M1) and “alternatively” (M2) activated microglia [15, 30] (Figure 1). This subdivision is based on function that while M1 microglia are often associated with acute infection, M2 cells play a role in tissue remodelling, repair, and healing [30]. However, M1 macrophages are also vital and important for the defence against microorganisms. The functional classification relies on the microenvironment of chemo- or cytokines as a result of microbial products or damaged cells [30]. IFN-*γ* and lipopolysaccharide (LPS) polarize microglia towards the M1 state and induce the release or expression of interleukin- (IL-) 1, IL-6, IL-12, IL-23, and inducible nitric oxide synthase (iNOS). On the other hand, the presence of IL-4, IL-10, and IL-13 turns microglia into M2 cells, which produce IL-10 and express arginase 1 [30, 31]. However, one has to keep in mind that this distinction into M1/M2 is a simplification and represents the extreme states. During disease both of these extremes as well as intermediate states may be present. However, the described subdivision of M1 and M2 cells nicely reflects the Janus-like behavior of microglia regarding their promotion of either tissue injury or repair. However, more and more evidence has accumulated that microglia do not only take part in immunological processes but also play a role in nonpathological conditions by, for example, eliminating or remodelling synapses and support of myelin turnover [32–34]. Among all the described functions of microglia, this review concentrates specifically on the involvement of microglia in autoimmune diseases of the CNS. In particular, we elucidate the mechanisms of microglia activation and the subsequent results of activation. Finally, we highlight current ideas to interfere in microglia activation as promising therapeutic strategies for MS. 

## 3. Function of Microglia in CNS Autoimmunity

### 3.1. Activation of Microglia as Hallmark of Disease

For several decades, the activation of microglia has been described in the damaged CNS during the pathology of MS/EAE, reflecting an initial event in MS pathology [35]. Even in early stages of MS, activated microglia clusters, so-called microglia nodules, are found in preactive lesions in the white matter of MS patients [36, 37]. In response of becoming activated, microglia also start to proliferate during MOG_35-55_-induced EAE [22]. In order to gain further insights into the involvement of activated microglia, a CD11b-*HSVTK *transgenic mouse line was generated [38]. In this mouse model the *HSVTK* gene is driven by the CD11b promoter, which is only expressed in cells with myeloid origin including microglia and macrophages. Importantly, *HSVTK* acts suicidal upon ganciclovir treatment. Thus, CD11b-*HSVTK* represents a pharmacogenetically inducible *in vivo* model of microglia depletion. MOG immunization of ganciclovir-treated CD11b-*HSVTK *mice significantly repressed disease onset and the severity of clinical EAE signs. The importance of microglia activation during EAE pathology was confirmed by application of macrophage inhibitory factor (TKP) or minocycline, which also attenuated EAE symptoms [39, 40]. Recently, the involvement of the complement system, which usually plays a part in defence and elimination of microorganisms in the adaptive immune response [41], in microglia activation in EAE was shown [42]. Under pathological conditions, like MS, a first disease-causing stimulus primes microglia, which upon a second stimulus result in overactivated microglia [42]. The presence of primed microglia was observed in MS lesions and in a knock-out of complement receptor-1-related protein y (Crry) [42]. During EAE primed microglia led to overactivated microglia resulting in an enhanced clinical severity in Crry-deficient mice [42]. Thus, microglia activation presents a histological hallmark in MS pathogenesis. However, during the disease, activated microglia might fill two positions depending on their polarization, M1 cells are involved in the pathogenic T-cell response (T_H_1 and T_H_17), while M2 microglia are designated as being protective in MS [43, 44]. Therefore, it is important to understand the processes and mechanisms of microglia activation and polarization. 

Recently, miRNA124 (miR-124) was identified to be a key regulator and modulator of microglia/monocyte activation [29]. miR-124, which is highly expressed in ramified microglia, belongs to the family of noncoding microRNAs, which are known to have regulatory functions such as promoting mRNA degeneration or interfering with mRNA translation [29, 45]. Interestingly, while during onset and peak of EAE expression of the surface markers CD45 and MHC class II was upregulated in microglia, the expression of miR-124 was repressed [29]. miR-124 targets C/EBP-*α* and thereby decreases the expression of C/EBP-*α* and indirectly Pu.1, both important myeloid regulatory transcription factors responsible for expression of activation markers [29]. Finally, intravenous treatment of miR-124 in the preclinical phase as well as after onset of the disease inhibited or substantially ameliorated the clinical course of EAE. Importantly, application of miR-124 turns microglia towards an M2 polarization. 

In addition, suppressor of cytokine signalling (SOCS-) 3 was identified as another key player of microglia activation [31]. SOCS proteins are known to interfere with JAK/STAT signalling. SOCS3 in particular inhibits IL-6 signalling by limiting STAT3 activation [46]. Induction of EAE in the absence of SOCS3 on myeloid cells, by using LysMCre-SOCS3^fl/fl^ mice, resulted in an early onset and more severe disease course, with an overly activated STAT3/4 signalling [31]. Myeloid-specific SOCS3 deficiency polarized the microglia/monocytes towards the M1 state and induced neuronal death. Thus, SOCS3 limits activation of macrophages/microglia and controls their polarization status. Taken together, both, miR-124 as well as SOCS3, are important factors critical for microglia activation. The hereby activated microglia then take over several responsibilities depending on their polarization.

### 3.2. Microglia as a Source of Cytokines during Autoimmune Inflammation

Cytokines and chemokines are small secreted signalling molecules important for inter- and intracellular communication during inflammation. While both are mostly expressed at very low, basal levels in healthy conditions, the expression and secretion, primarily by microglia but also by astrocytes, are markedly increased upon CNS insult [47–50]. Microglial secretion of diverse cytokines can modulate cells in a paracrine manner but can also affect microglia in an autocrine fashion by either positive or negative feedback loops [47, 50]. Finally, the amount and composition of cytokines and chemokines in the microenvironment determines the function of microglia as indicated earlier in description of M1/M2 microglia polarization. 

 IFN-*γ*, tumour necrosis factor- (TNF-) *α*, IL-1*β*, and IL-6 are potent proinflammatory cytokines activating microglia and shifting them towards the cytotoxic M1 phenotype, thereby potentiating the inflammatory response [49–52] (Figure 1). However, to induce the complete cytotoxic M1 state, which leads to the expression of TNF-*α*, IL-1*β*, IL-2, IL-6, and IL-12, as well as iNOS and cyclooxygenase-2 (COX-2), binding of more than one cytokine is necessary [50]. Successful stimulation then triggers the expression of target genes via various signalling cascades including NF-*κ*B, JNK, ERK1/2, and p38 [50, 53, 54]. Subsequently, constitutive activation of, for example, the NF-*κ*B signalling cascade in microglia/macrophages in LysMCreI*κ*B*α* ^fl/fl^ mice worsens clinical symptoms of EAE and increases the expression of, for example, IL-1*β* and IL-6 [55]. Interestingly, NF-*κ*B signalling is not only important in microglia, but also plays an important role in neuroectodermal-derived astrocytes during autoimmune inflammation of the CNS [56, 57]. 

The roles of the proinflammatory cytokines IL-12, IL-23, and IL-17 produced by classically activated M1 microglia are more sophisticated [58–60] (Figure 1). Interestingly, IL-12 and IL-23 belong to the IL-12 family of cytokines and share a similar *β*-chain p40 but differ in their *α*-chain, which is p19 for IL-23 and p35 for IL-12 [60]. Their contribution to the pathogenesis of EAE lies in the regulation, proliferation, and differentiation of naïve CD4^+^ T cells. While IL-12 facilitates T_H_1 effector cell differentiation, IL-23 is critical for stable IL-17 expression which is ultimately important for the differentiation of pathogenic T_H_17 cells [50, 60, 61]. Although T_H_1 and T_H_17 both hold central and distinct roles in the development and pathogenesis in CNS autoimmune inflammation, their exact functions remain to be fully resolved [61]. In contrast, alternatively activated M2 microglia express and release the cytokines IL-4 and IL-10, as well as transforming growth factor- (TGF-) *β* through which the differentiation of anti-inflammatory and protective T_H_2 and T regulatory (T_reg_) cells is induced [61]. 

 In addition to cytokines, microglia are also activated by and are able to release diverse chemoattractant chemokines, such as CCL2, CCL3, CCL4, CCL5, CXCL10, and/or CCL12 [47, 48, 52, 62, 63] (Figure 1). The importance of CCL2, also known as monocyte chemoattractant protein- (MCP-) 1, and its receptor CCR2 has been investigated for various CNS pathologies such as neurodegeneration [23] and autoimmune inflammation [63–65]. Many different proinflammatory stimuli, such as IFN-*γ*, TNF-*α*, and IL-1*β*, can induce the expression of CCL2 in the brain [63]. Until now, two major functions have been allocated to CCL2 during CNS autoimmunity. First, it participates in the disruption of blood brain barrier integrity. Secondly it is involved in the recruitment of CCR2+CD11b+Ly-6C^hi^ mononuclear leukocytes into the CNS [63, 64, 66]. Consequently, deletion or blockade of CCL2 synthesis by the chemical inhibitors significantly reduced the clinical symptoms of EAE [67–70]. In addition, therapeutic interference with the function of CCL5/Rantes reduced leukocyte activation and trafficking to the CNS [68]. In summary, by arranging the cyto-/chemokine milieu, microglia play a key role both in regulating and recruiting leukocytes into the CNS as well as macrophage polarization during autoimmune inflammation [71].

### 3.3. Phagocytosis Mediated by Microglia: Pathfinding for Reengineering

Phagocytosis can be seen as a double-edged sword; on the one hand, it is beneficial by clearing cellular debris, but on other the hand it can also be destructive by inducing an oxidative burst [72]. Microglia, as macrophages, are per definition phagocytes of either invading pathogens or dead cells in the CNS [72, 73]. During EAE, activated microglia are known to phagocytize myelin debris and oligodendrocytes in lesions, which in turn leads to the release of proinflammatory cytokines [71, 73] (Figure 1). One of the key receptors involved in this process during EAE is the triggering receptor expressed on myeloid cells (TREM-) 2 together with its associated signalling molecule DNAX-activating protein- (DAP-) 12, both expressed in microglia and macrophages [74, 75]. Virus-mediated overexpression of TREM2 in myeloid cells increased the clearance of myelin debris, thereby improving the tissue regeneration and consequently reducing the severity of clinical symptoms [75]. In addition to TREM2, several other receptors are important for myelin debris phagocytosis, such as complement receptor (CR) 3, signal regulatory protein (SIRP)-*α*, or Fc*γ* receptor [72]. 

Interestingly, remaining myelin debris inhibits the regenerative remyelination, indicated by a loss of *PDGFR*α**+ oligodendrocyte precursor recruitment [76, 77]. A detailed analysis, making use of gene arrays, could determine a “single remyelination-supportive microglia” phenotype, which gradually develops [78]. Additionally, during remyelination microglia express several genes, which are known to promote oligodendrocyte differentiation [78]. Thus, efficient resolution of inflammation, by phagocytosis of myelin debris, is important for tissue reengineering. 

### 3.4. Antigen Presentation and T Cell Priming by Microglia

MS/EAE pathology is characterized by harmful T cell infiltration [5]. For T cell activation by an antigen presenting cell (APC), two signals are prerequisites: first, antigen peptide presentation at the cell surface via major histocompatibility complex (MHC) to the specific T cell receptor [79, 80] and, second, a costimulatory interaction of CD80/86 or CD40 located on APCs with CD28 or CD40L present on T cells [71, 81]. Typically, dendritic cells represent the most prominent cell type for antigen presentation [82]. However, in addition to T cell attraction, by expressing CCL2 and CCL5, microglia can also serve as APCs by presenting myelin [83]. During MS/EAE pathology, microglia highly express MHC class I and II proteins as well as CD40 and CD80/86 [48, 84] (Figure 1). Microglia “reprime” or reactivate T cells in lesion sites and thereby exacerbate the disease by epitope spreading [71, 85, 86]. Following the T cell-APC interaction, differentiation into mature proinflammatory encephalitogenic T cells (T_H_17, T_H_1) or anti-inflammatory T cells (T_H_2 or T_reg_) takes place, depending on the cytokine environment [71]. 

### 3.5. Microglia as Therapeutic Targets

With the increased understanding of microglia function in MS/EAE pathology gained thus far, it might be possible to find and establish efficient therapies by targeting microglial responses. Since M1 microglia seem to be harmful during CNS autoimmune inflammation, most therapeutic ideas aim to either deactivate M1 microglia or turn them into M2 cells. Recently, galectin- (gal-) 1 was identified as a potent microglia regulator [87]. Gal-1 preferentially binds to M1 microglia, thereby modulating M1 key features, such as CCL2 and iNOS expression, by controlling NF-*κ*B and p38 signalling [87]. Finally, gal-1 application ameliorated disease course of EAE, making it a potentially attractive therapeutic option [87]. Similar approaches were employed using tuftsin, a naturally occurring tetrapeptide known to induce phagocytosis, or ghrelin, which acts as an anti-inflammatory hormone [88, 89]. Treatment with tuftsin or ghrelin shifted microglia into M2 polarization *in vitro* and reduced EAE severity *in vivo* [88, 89]. In addition, application of compound A, a plant-derived phenyl aziridine precursor, ameliorated the clinical course of EAE, and subsequent *in vitro* analyses revealed an inhibition of NF-*κ*B signalling by compound A in microglia [90]. *β*-Lapachone treatment, a natural substance from the bark of the lapacho tree, significantly reduced the expression of IL-12 family cytokines in microglia and thereby suppressed clinical severity [91]. 

 During the termination of the acute inflammatory reaction, microglia become deactivated [15]. Therefore, the deactivation of microglia also presents a promising therapeutic concept. Several molecules, including chemokines and steroid hormones, are known as ligands regulating microglia activation status and mediate signalling via different nuclear receptors [15]. In this respect, stimulation of the oestrogen receptor- (ER-) *β* with synthetic ligands efficiently turned down microglia activation and thereby reduced EAE symptoms [92, 93]. 

Recently, the use of reprogrammed embryonic stem (ES) cell-derived microglia-like cells (ESdM) for MS was suggested [94]. Intravenously applied ESdM populate lesion sites in the spinal cord of EAE mice but do not influence the clinical course [94]. Application of lentiviral transduced ESdM with NT-3, a neurotrophic factor, significantly reduced clinical disease severity as well as the degree of demyelination and axonal damage [94]. 

## 4. Conclusion

Activation of microglia is a hallmark of MS/EAE pathology. During recent years more and more knowledge about how and why microglia become activated has accumulated. Subsequently, several investigations have already proven the feasibility of beneficially modulating microglia function in animal models of MS. Nevertheless, this path has to be taken further. There is a need for comprehensive and detailed analysis to further illuminate the triggering and signalling mechanisms of microglia in inflammation. Thoroughly understanding microglia action in the context of a disease will help us identify new targets for therapeutic approaches, which may ultimately be translated to the clinics.
